# Management of critically ill patients following earthquake disasters

**DOI:** 10.55730/1300-0144.6033

**Published:** 2025-04-22

**Authors:** Esat Kıvanç KAYA, Burçin HALAÇLI, Mehmet YILDIRIM, Arzu TOPELİ

**Affiliations:** Division of Intensive Care Medicine, Department of Internal Medicine, Faculty of Medicine, Hacettepe University, Ankara, Turkiye

**Keywords:** Earthquake, disaster, crush syndrome, trauma, intensive care, critically ill

## Abstract

The devastating earthquakes that struck southeastern Türkiye on February 6, 2023, underscored the profound social and healthcare challenges of large-scale natural disasters. These events highlighted the need for healthcare professionals to be well-prepared and equipped with the expertise to manage such crises effectively. Earthquake-induced trauma presents a wide array of medical and surgical complications, frequently requiring urgent hospitalization and specialized management for critically ill patients. However, the limited availability of comprehensive data and evidence-based guidelines complicates optimal care delivery in these scenarios. This review outlines current recommendations for the multidisciplinary management of critically ill patients with earthquake-related trauma. It underscores the significance of promptly identifying and addressing acute complications, including crush syndrome, acute kidney injury, infections, and sepsis. Key strategies such as individualized fluid and electrolyte management, nutritional interventions, and rigorous infection control measures are discussed. Additionally, this review highlights the critical roles of physiotherapy and psychological support in the rehabilitation process, as well as the importance of long-term follow-up for survivors at risk of postintensive care syndrome and posttraumatic stress disorder. To enhance future disaster response and patient outcomes, the review calls for strengthened collaboration, preparedness, and research efforts. This comprehensive examination provides a framework for managing critically ill patients hospitalized due to earthquake-related trauma, offering valuable insights for healthcare providers across multiple disciplines.

## 1. Introduction

On February 6, 2023, at 04:17 and 13:24 local time, two consecutive earthquakes with magnitudes of 7.7 and 7.6, respectively, on the Richter scale and an intensity exceeding 11 struck the southeastern Türkiye, home to over 13 million people. The epicenter was Kahramanmaraş Province. These catastrophic earthquakes affected an area spanning over 300 km and impacted 11 major provinces. According to official reports, more than 44,000 individuals lost their lives, and approximately 120,000 were injured.

Although Türkiye is no stranger to natural disasters, these earthquakes placed an unprecedented strain on the healthcare system due to their magnitude, sequential occurrence, and widespread disruption of infrastructure. Given the large number of injured individuals, ensuring optimal management of critically ill patients requiring immediate hospitalization for earthquake-related trauma is essential for improving outcomes.

Earthquakes can cause a wide range of medical and surgical complications affecting nearly all organ systems. However, the lack of robust data and evidence-based guidelines poses significant challenges for healthcare providers, including internists, intensivists, infectious disease specialists, nephrologists, and trauma surgeons, who must manage these patients [1]. This review aims to present current recommendations for the management principles of critically ill patients hospitalized due to earthquake-related trauma.

## 2. Definitions and associated pathophysiological mechanisms

### 2.1. Crush injury and rhabdomyolysis

Crush injury refers to tissue trauma and ischemia-reperfusion injury resulting from trauma to the thorax, extremities, or other body regions [2]. Following muscle necrosis, releasing intramuscular molecules such as lactic acid, creatine kinase (CK), creatinine, myoglobin, phosphate, and potassium into the circulation leads to rhabdomyolysis [3]. The classic triad of rhabdomyolysis symptoms includes muscle pain, weakness, and dark-colored urine resembling cola. A CK level exceeding five times the upper limit of normal or >1000 IU/L confirms the diagnosis [4].

Muscles, accounting for approximately 40% of total body mass, can be considered the largest organ system in the body and are particularly vulnerable to trauma. Muscles are enclosed by noncompliant, rigid fascia that form compartments. Together with the sarcolemma, these compartments create a liquid-proof structure [5]. When muscles are subjected to external compression, sarcolemma function deteriorates, leading to electrolyte shifts: calcium, sodium, and water move intracellularly, while potassium and myoglobin shift extracellularly [6]. Accumulation of intracellular free calcium triggers mitochondrial damage, activation of proteases and phospholipases, and depletion of adenosine triphosphate (ATP) stores [7]. In ischemic tissue injury caused by major compression, the most significant damage occurs after reperfusion. When oxygen becomes available during reperfusion, free oxygen radicals are generated as leukocytes migrate into the damaged tissue [5]. This phenomenon, known as ischemia-reperfusion injury, may exacerbate tissue damage, potentially resulting in sepsis, acute kidney injury (AKI), multiorgan failure (MOF), or acute respiratory distress syndrome (ARDS) [8].

### 2.2. Compartment syndrome

Compartment syndrome arises from tissue and nerve damage caused by impaired circulation and increased intracompartmental pressure due to muscle edema and swelling. Pain begins when intracompartmental pressure reaches 20–25 mmHg, and capillary blood flow becomes compromised at 25–30 mmHg. A delta pressure (diastolic blood pressure minus intracompartmental pressure) of <30 mmHg indicates acute compartment syndrome, warranting immediate evaluation for fasciotomy based on clinical findings [9,10]. Comprehensive examination, including palpation of the body for pain, pressure, paresthesia, paresis/paralysis, paleness, and pulselessness, is crucial [9].

### 2.3. Crush syndrome

The systemic complications following crush injury, particularly those progressing to organ dysfunction (e.g., AKI or MOF), are termed crush syndrome (CS) [11]. During crush injury, fluid shifts from the extracellular to intracellular compartments within muscles, leading to depletion of intravascular volume, causing renal hypoperfusion and ischemia. This results in prerenal AKI, which can progress to acute tubular necrosis (ATN) if not managed appropriately [6]. Myoglobinuria contributes to AKI through intratubular cast formation [7]. Additional complications include hyperkalemia, hyperphosphatemia, and hypocalcemia that exacerbate renal hypoperfusion and impair myocardial contractility and cardiac output [5].

### 2.4. Trauma-induced coagulopathy

**Figure 1:**
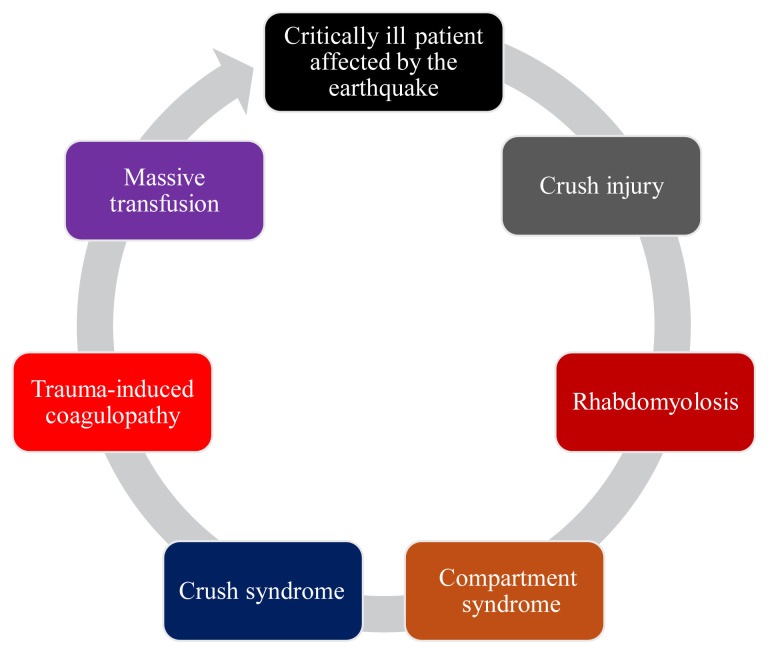
The cycle of critically ill patients affected by the earthquake.

Trauma-induced coagulopathy refers to the disruption of coagulation and increased bleeding following vascular endothelial injury due to trauma and/or hemorrhagic shock. This creates a vicious cycle of bleeding and coagulopathy, aggravated by hypothermia, acidosis, and dilution of clotting factors during massive transfusion. Over time, coagulation abnormalities may lead to hypercoagulability and microthrombus formation, ultimately resulting in organ failure [12]. Massive transfusion is defined as the need for 10 units of red blood cells (RBCs) within 24 h, 3 units of RBCs within 1 h, or 4 units of any blood product within 30 min. A 1:1:1 ratio of RBCs, plasma, and platelets is recommended for replacement [13]. However, complications of massive transfusion may include disseminated intravascular coagulation (DIC), hypothermia, hyperkalemia (due to stored blood products), and hypocalcemia, as well as metabolic acidosis or alkalosis due to citrate accumulation [14] (Figure 1).

## 3. Epidemiology

The determinants of injury during earthquake disasters are multifactorial. Factors include the magnitude of the earthquake, the affected region, population density (urban versus rural), the structural integrity of buildings, the timing and season (influencing body temperature), the duration of entrapment, the efficiency of rescue operations, and the characteristics of the healthcare system [15]. However, due to the lack of detailed comparative data and medical evidence, the short- and long-term outcomes of earthquake victims remain poorly understood.

Typically, 80% of individuals trapped under rubble die immediately, while 10% survive with minor injuries. The remaining 10% suffer severe injuries requiring hospitalization, including admission to intensive care units (ICUs) [16]. Data from the Marmara earthquake, involving nearly 10,000 patients, revealed that 53.9% required hospitalization in reference hospitals, with an overall mortality rate of 4.3% [1]. An epidemiological study of earthquakes in developing countries indicated that most injuries requiring treatment (87%) were orthopedic, with fractures accounting for 65%. The most common fracture sites were the tibia/fibula (27%), femur (17%), and foot/ankle (16%). Approximately 42% of injuries involved multiple fractures, 22% were open fractures, and 16% were comminuted fractures [17]. Patients admitted within the first 24 h postearthquake typically had more severe injuries and complications compared to those admitted later [18].

Crush injuries are observed in 3%–20% of earthquake-related trauma cases [19]. Traumatic rhabdomyolysis and CS are among the most life-threatening complications following earthquakes. AKI occurs in approximately 30%–50% of patients with rhabdomyolysis [20]. During the Tangshan earthquake in Hebei Province, China, CS was reported in 2%–5% of the nearly 400,000 injured patients [21]. Analysis of the Kobe earthquake in Japan showed that 50% of patients with CS developed AKI, and half of these required dialysis [22]. Predictors of AKI in crush injuries include prolonged entrapment, multiple crush injuries, male sex, infection, and elevated CK levels [23]. A study from Iran corroborated these findings, identifying entrapment duration and elevated CK levels as significant predictors of AKI, alongside lactate dehydrogenase (LDH), aspartate aminotransferase (AST), and uric acid [24]. Mortality rates for CS patients requiring dialysis were 41% in the Kobe earthquake but lower (15%–20%) in the Marmara (Türkiye), Chi-Chi (Taiwan), and Kashmir (Pakistan) earthquakes [25].

In addition to AKI, other major complications include infection, sepsis, hypovolemic shock, ARDS, compartment syndrome, DIC, bleeding, cardiac failure, arrhythmia, and electrolyte disturbances [26]. Many of these cases necessitate ICU admission, although the exact frequency of ICU requirements remains unclear. A Chinese epidemiological study reported ICU admission in 3.3% of nearly 33,000 hospitalized cases. Factors independently associated with mortality include severe traumatic brain injury (TBI), MOF, CS, cardiac or respiratory disease, advanced age (≥65 years), and the need for ICU care [27]. A recent multicenter study involving 201 critically ill patients admitted to 12 ICUs following the Kahramanmaraş earthquakes reported an ICU mortality rate of 10%. Independent risk factors associated with mortality included the presence of CS, advanced age, vasopressor therapy, and a lower Glasgow Coma Scale (GCS) score [28].

Data from the Marmara earthquake revealed that, among 639 (12%) of over 5000 hospitalized patients with renal complications, 34.9% developed infections, 18.9% developed sepsis, 9.8% experienced cardiovascular complications, 7.4% had ARDS, and 6.8% developed DIC. Additionally, 9% required renal replacement therapy (RRT), and 3.4% required invasive mechanical ventilation [1]. Complications such as massive transfusion, sepsis, oxygen toxicity, pneumonia, and DIC can also lead to ARDS [29]. A retrospective study of Marmara earthquake patients found that DIC (odds ratio: 5.8) and ARDS (odds ratio: 4.5) were independent predictors of mortality [30].

Fasciotomy was performed in almost half of the patients with compartment syndrome in the Marmara earthquake, a higher rate than the 13.1% reported during the Kobe earthquake [22]. In the 2003 Bingöl earthquake in Türkiye, 70% of patients with compartment syndrome underwent fasciotomy, with 81% subsequently developing sepsis due to wound infections [31]. Retrospective data from the Bam earthquake in Iran indicated that 35% of patients required fasciotomy. Notably, fasciotomy did not worsen morbidity or mortality in patients with crush injury-related AKI [32]. Timely and appropriate intensive care support has been shown to improve outcomes in patients with compartment syndrome [33].

## 4. Follow-up parameters and therapeutic approaches

Critically ill trauma patients affected by earthquake disasters require meticulous monitoring. Key laboratory parameters, including electrolytes, venous blood gas (with a focus on lactate and base excess), and complete blood count (CBC), should be assessed every 6–12 h, depending on the time elapsed since hospital admission. We recommend to calculate the strong ion difference (SID) [Formula = (sodium + potassium) − (chloride + lactate)] and corrected anion gap [Formula = sodium − (chloride + bicarbonate); for each 1 g/dL below 4 g/dL, 2.5 mEq is added to the anion gap value for low albumin] every 12–24 h [34,35]. For patients with urine output, urine color should be closely observed, and urinalysis for density and pH should be conducted to monitor renal function. Daily and postdialysis levels of blood urea nitrogen (BUN), creatinine, albumin, uric acid, CK, myoglobin, LDH, AST, ALT, total bilirubin, international normalized ratio (INR), activated partial thromboplastin time (aPTT), fibrinogen, and D-dimer (for DIC workup) should also be monitored [4]. Inflammatory markers such as C-reactive protein (CRP), procalcitonin (PCT), and prealbumin should be measured upon admission and periodically thereafter as indicated [36]. Hourly monitoring of total fluid administration and fluid loss is crucial to maintain optimal fluid balance. A thorough evaluation of the six P’s (pain, pressure, paresthesia, paresis/paralysis, paleness, and pulselessness) should be performed on affected extremities every 12 h [5]. Additionally, daily neurological examinations should be conducted, and body and extremity temperatures should be checked to detect any signs of deterioration or complications. Trauma imaging should be completed for all patients transferred from external facilities to ensure a comprehensive assessment of injuries.

### 4.1. Fluid therapy

Considering the pathophysiology of rhabdomyolysis and CS described above, prompt and appropriate fluid resuscitation is critical in preventing the occurrence of CS and its renal complications. For example, the need for dialysis in crush injury patients from the 2003 Bingöl, Türkiye, earthquake was notably lower compared to other disasters during the same period, likely due to early and vigorous fluid resuscitation [31]. Similarly, intensive fluid administration during the Haiti earthquake was shown to reduce the likelihood of requiring dialysis [37]. However, a one-size-fits-all approach to fluid resuscitation does not apply to earthquake-related traumas. Several factors must be considered for fluid replacement, including the extent of injury, body surface area, environmental and body temperature, urine output, age, and preexisting conditions such as heart failure or chronic kidney disease (CKD).

Patients rescued after prolonged entrapment may require higher fluid volumes, whereas a more conservative approach may be warranted in those with anuria [38]. The Marmara earthquake experience demonstrated that patients who required dialysis often received excessive fluids, potentially due to hypervolemia in those admitted several days after the disaster [39]. Risk factors for volume overload include advanced age, younger age, low body mass, and mild trauma. Fluid administration should be limited in patients at high risk of volume overload, as well as in individuals trapped under rubble in low-temperature environments [38]. Intravenous (IV) fluids should be initiated as soon as possible, ideally even while the victim is still trapped, at a rate of 500–1000 mL/h (10–15 mL/kg) [25]. The initial infusion rate can be reduced by at least 50% after 2 h, followed by reassessment at 6-h intervals. In the presence of anuria, fluid replacement may continue at 500–1000 mL/day, adjusted to include daily insensible fluid losses. For cases where close monitoring is challenging during mass disasters, fluid intake should be limited to 3–6 L/day.

Crystalloids containing potassium should be avoided during initial resuscitation due to the risk of undetected hyperkalemia. Balanced crystalloids may be preferred for maintenance fluids based on the patient’s potassium levels. For patients with hypernatremia or hyperchloremia, balanced crystalloids are recommended, as vigorous isotonic saline replacement can increase the risk of hyperchloremic acidosis and AKI [40]. Colloids are not recommended for fluid resuscitation in earthquake victims [41].

IV fluids should be reduced and discontinued once oral intake is feasible. In cases involving burns or similar skin injuries, fluid resuscitation can follow the Parkland formula [4 mL × kg × burn percentage], with half the volume administered in the first 8 h and the remainder over the following 16 h [42]. A target urine output of 1–3 mL/kg/h is desirable, and a higher output of up to 300 mL/h may be acceptable in specific cases [4]. If hypervolemia develops after clinical recovery, excess fluid should be actively removed.

### 4.2. Diuretics

Mannitol diuretic, antioxidant, and vasodilatory properties emphasize its potential as an adjunct in specific clinical settings. While these effects may help lower intracompartmental pressure, alleviate muscle edema and pain, and prevent renal tubular cast formation, their routine use in CS-associated AKI is not recommended due to limited evidence [43,44]. Mannitol may be included as part of IV fluid therapy, but should not be used independently. It is contraindicated in patients with oligoanuria but may be considered in those with urine output exceeding 20 mL/h. The recommended dosage is 1–2 g/kg/day, administered at a maximum rate of 5 g/h, with a total daily limit of 120 g [6]. If urine output fails to reach 30–50 mL/h, mannitol therapy should be discontinued [45]. To minimize the risk of hyperosmolarity and electrolyte imbalances during repeated use, sodium levels should remain below 150 mEq/L, and serum osmolality should stay under 320 mOsm/kg [46].

Studies on loop diuretics in AKI patients have shown no significant survival benefit, although they may shorten the oliguric phase and potentially decrease the need for dialysis [47]. These diuretics can reduce oxygen consumption and metabolic demand in proximal tubular cells, which is considered a beneficial effect. However, they also have notable limitations, such as increasing urinary pH, promoting afferent arteriole vasoconstriction, and contributing to the accumulation of heme proteins in the tubular lumen [4]. Consequently, routine use of loop diuretics in AKI associated with earthquake-related injuries is not advised. Nonetheless, their use may be appropriate in specific scenarios, including in hypervolemic or elderly patients and those who are fluid-resuscitated and at risk of overhydration, especially if they develop ARDS [41].

### 4.3. Electrolyte imbalances

Hyperkalemia is a hallmark feature and a major contributor to morbidity and mortality. This condition can cause life-threatening arrhythmias if not promptly addressed. Hyperkalemia often necessitates immediate intervention, including calcium (calcium gluconate or calcium chloride) administration for cardiac protection, insulin and glucose therapy, inhaled short-acting beta-2 adrenergic agents, and potentially RRT [48].

Hypocalcemia commonly occurs in the acute phase due to the precipitation of calcium with released phosphate from necrotic muscle tissue. However, hypercalcemia may develop during the recovery phase as calcium is mobilized back into circulation. Hyperphosphatemia results from the efflux of phosphate from lysed cells, exacerbating hypocalcemia through calcium-phosphate precipitation and deposition in tissues [4].

### 4.4. Metabolic acidosis

AKI associated with earthquake-induced rhabdomyolysis can be further aggravated by acidic urine [10]. While randomized controlled trials are unavailable, data from small case series and retrospective studies suggest that urine alkalization may reduce renal damage in rhabdomyolysis secondary to CS and could potentially improve AKI outcomes [4].

The primary purpose of administering sodium bicarbonate is to increase urine pH above 6.5, thereby inhibiting the accumulation of heme proteins in the renal tubules. Moreover, alkalization can aid in correcting hyperkalemia and metabolic acidosis. When combined with isotonic saline, sodium bicarbonate may also prevent hyperchloremic metabolic acidosis. In the absence of alkalosis, 200–300 mEq of sodium bicarbonate can be administered over the first 24 h [17]. Blood pH and bicarbonate levels should be closely monitored to ensure they remain below 7.5 and 30 mEq/L, respectively.

The use of sodium bicarbonate carries potential risks, including calcium phosphate precipitation, hypernatremia, hypocalcemia, and hypervolemia, necessitating regular assessment of ionized calcium levels [45]. Hypocalcemia can worsen the cardiac effects of hyperkalemia, making calcium therapy a critical consideration. Calcium should be administered not only in cases of symptomatic hypocalcemia but also empirically for cardiac protection during severe hyperkalemia, especially when laboratory facilities are limited. The effects of calcium are transient, lasting approximately 30–60 min, and may require repeated doses. During the recovery phase of crush injuries, calcium sequestration in muscle tissue can lead to hypercalcemia, which must be considered when planning calcium supplementation [38].

### 4.5. Renal replacement therapy

The indications for RRT in this context align with standard clinical practice. These are severe hyperkalemia (>7 mmol/L), metabolic acidosis (pH <7.1), significant uremia (BUN >100 mg/dL or creatinine >8 mg/dL) and its complications, as well as oligoanuria accompanied by hypervolemia. Prophylactic or early initiation of dialysis may also be considered in patients with rapidly escalating potassium levels [45]. Intermittent hemodialysis (IHD) is recommended as the initial RRT modality due to its medical efficacy and logistical feasibility [49]. While peritoneal dialysis (PD) has been attempted in a limited number of cases during past earthquakes, its application in adults is generally unsuitable owing to its slower action and suboptimal potassium clearance [39].

Extracorporeal methods for myoglobin removal have been proposed and investigated in patients with AKI to mitigate nephrotoxicity [50]. Given that myoglobin has a molecular weight of 17 kDa and low protein-binding affinity, it is inadequately removed via diffusion during conventional IHD. Instead, its elimination predominantly relies on convection (ultrafiltration). Studies have demonstrated that continuous veno-venous hemofiltration (CVVH) can remove significant amounts of myoglobin [51]. However, these studies involved small patient populations and provided insufficient evidence to establish definitive guidelines. Furthermore, CVVH is less effective than IHD in addressing severe hyperkalemia [49].

In patients with hemodynamic instability, continuous renal replacement therapy (CRRT) may be considered, particularly in cases of ischemia-reperfusion injury that can induce a hyperinflammatory state and exacerbate hemodynamic compromise. CRRT may also facilitate cytokine removal in such scenarios, although the evidence supporting this approach remains limited [52].

### 4.6. Infections

Infections associated with CS have been reported with incidences ranging from 25.8% to 67.2% [53]. The primary sources of infections are often wounds resulting from crush injuries and fasciotomy sites [54]. Given that nearly one-third of patients with CS may develop infections and one-fifth may progress to sepsis, implementing robust infection control measures is critically important [1]. The most common pathogens causing sepsis in CS cases include *Acinetobacter* spp., *Pseudomonas* spp., *Klebsiella* spp., *Enterobacter* spp., and *Staphylococcus aureus*. In a recent multicenter study conducted in patients who suffered from the 2023 Kahramanmaraş earthquakes, the most frequently isolated pathogens in patients admitted to the ICU due to earthquake-related injuries were identified as *Acinetobacter baumannii* (16.4%), *Klebsiella pneumoniae* (16.4%), *Enterococcus* spp. (10.5%), *Escherichia coli* (10.5%), and *Pseudomonas aeruginosa* (9.9%) [55].

To mitigate this risk, strict adherence to infection control protocols is essential. Measures such as maintaining hand hygiene, ensuring proper wound care, managing catheters and drains, and implementing appropriate isolation precautions for infected patients are paramount [56]. In cases involving penetrating traumas to the head, chest, and abdomen, as well as fasciotomy, skull fractures, open facial fractures, and other fractures, prophylactic and preemptive antibiotic therapy may be warranted [45]. Based on prior earthquake-related experiences, the etiological factors of infections, and patterns of antibiotic resistance [56], our recommendations for antibiotic prophylaxis and preemptive treatment are detailed in Table 1. Moreover, the pathogens isolated following major earthquakes are often predominantly multidrug-resistant (MDR). Therefore, it is crucial to tailor antibiotic therapy based on the patient’s condition and risk factors, ensuring that MDR pathogens are also considered during adjustments [55].

Furthermore, all patients should be assessed for tetanus immunization. If there is no history or documentation of vaccination, a single dose of tetanus vaccine should be administered. In patients without a prior history of tetanus vaccination and presenting with iron-stinging injuries or wounds heavily contaminated with soil, antitetanus serum should be provided [57].

### 4.7. Analgesia

Proper pain management is critical for patients with crush injuries. As a first-line analgesic, paracetamol is advised, with a maximum daily dose of 4 g/day. This should be reduced to 2 g/day in individuals with hepatic dysfunction. The use of nonsteroidal antiinflammatory drugs (NSAIDs) is discouraged [45] due to their potential to cause renal vasoconstriction, impair renal blood flow, and exacerbate conditions such as prerenal AKI and ATN [58].

For alternative pain relief, options include tramadol, morphine (given subcutaneously or intravenously), and fentanyl (administered via patches or intravenously). However, opioids should be used cautiously because they risk causing gastrointestinal motility issues [59].

Ketamine serves as a powerful alternative, providing both anesthetic and analgesic effects. It offers several benefits, including rapid onset, short duration of action, and multiple administration routes such as oral, IV, rectal, or intramuscular. Moreover, it does not cause cardiovascular or respiratory depression [60]. For localized pain relief, regional block anesthesia may also be an effective method, depending on the trauma site [59].

### 4.8. Nutrition

Malnutrition in patients with AKI secondary to CS is a complex issue. The hypercatabolic state that accompanies CS significantly worsens nutritional status. The onset of AKI in CS patients triggers various proinflammatory and prooxidative responses, including anorexia and impaired protein metabolism. An increase in nonspecific inflammatory biomarkers like CRP, interleukin-6 (IL-6), prealbumin, and transferrin are detected. Furthermore, factors such as insulin resistance, fluid overload, metabolic acidosis, and nutrient loss through dialysis filters can further exacerbate malnutrition. As a result, the loss of lean body mass cannot be attributed solely to inadequate nutritional intake during prolonged entrapment under rubble after an earthquake [61].

In the presence of hyperkalemia, a low-potassium diet is recommended. Refeeding syndrome poses a risk in patients who have undergone prolonged starvation due to extended entrapment. To avoid this complication, rapid refeeding with high-calorie intake should be prevented. Instead, supplementation with thiamine, multivitamins, and trace elements should be implemented daily, alongside careful monitoring of electrolyte levels. Nutritional support should be aimed at providing 30–35 kcal/kg/day of energy and 1.5–2 g/kg/day of protein for patients receiving IHD or CRRT [62].

### 4.9. Physiotherapy, psychological, and social supports

In earthquake-related trauma patients, prolonged hospitalization is often required, making rehabilitation a vital part of recovery once clinical stability has been achieved. Early mobilization is essential and should be conducted according to the guidelines of the orthopedic or surgical teams for patients who have undergone procedures like fasciotomy, debridement, or amputation due to trauma. It is important to make daily efforts to assist patients in getting out of bed, along with performing range of motion exercises and respiratory physiotherapy. These precautions avoid complications such as atelectasis, which can be caused by pain.

Psychiatric issues are commonly seen among earthquake survivors. Acute stress disorder often manifests within the first month following the disaster, with an increased risk of suicide attempts, making it critical to ensure patients are not left alone. Posttraumatic stress disorder (PTSD) may persist for longer than a month, and prolonged grief is also a potential issue. These patients are additionally more susceptible to delirium. To manage these concerns, psychiatric and social service consultations should be sought for every patient [45].

Extended hospitalization in earthquake survivors can lead to secondary complications in addition to the acute trauma-related conditions [63]. Persistent organ failure during prolonged hospital stays is referred to as chronic critical illness (CCI) [64]. For patients with MOF due to earthquake-related CS, CCI is a likely outcome.

Patients discharged after intensive care treatment may experience postintensive care syndrome (PICS), which involves persistent physical, cognitive, and psychological difficulties. These individuals need thorough follow-up care, including assessments of their quality of life and continued physiotherapy. Ongoing monitoring for PTSD is also essential.

Additionally, it is crucial to acknowledge that similar challenges, including burnout, may affect the families or caregivers of these patients. This phenomenon, known as PICS-family, requires evaluation and support [65]. A summary of management principles for critically ill patients affected by earthquake disasters is provided in Table 2 [66].

## 5. Future directions

Various drugs and medications have been proposed and studied in animal models for the prevention and treatment of earthquake-related CS [67]. However, no scientifically validated medication exists to specifically prevent or treat CS apart from optimal supportive therapies. The inherent challenges in conducting high-quality evidence-based studies in earthquake scenarios, compared to other critically ill patient groups, contribute to this gap. To advance understanding and treatment, it is essential to collect epidemiological data during disasters, enabling subsequent evaluations of applied therapies and consideration of regional, national, and institutional differences.

The devastating earthquake in Türkiye has underscored the importance of equipping all clinicians who manage critically ill patients with adequate disaster medicine training. Effective disaster response requires ongoing preparation, well-structured disaster management programs, and advanced training. Regular interdisciplinary meetings to discuss and refine treatment strategies should be organized for all physicians involved in critical care during such crises [25].

A significant challenge in disaster areas is the shortage of administrative and medical manpower. Rational allocation of healthcare professionals—whether already present in the affected area or arriving as volunteers—is vital to prevent burnout. In recent years, telemedicine has emerged as a valuable tool for remote patient monitoring and guidance, offering potential improvements in patient triage, monitoring, and specialist access [68].

A narrative review from 2014, encompassing 19 studies conducted in both real-life and simulated disaster scenarios, highlighted the effectiveness of telemedicine in facilitating communication between humanitarian aid workers and remote expert physicians for disaster management [69]. Similarly, a review by Rolston and Meltzer [70] emphasized the role of telemedicine in coordinating and integrating diverse organizations during unanticipated disasters. A recent scoping review by Sellers et al. [71] also outlines practical considerations in implementing disaster crisis standards of care within ICU settings, highlighting the need for predefined protocols, triage frameworks, and coordinated resource allocation to optimize response efforts during mass critical illness events. However, the use of telemedicine in the immediate aftermath of a severe disaster may be hindered by disruptions in electricity or telecommunications infrastructure [25]. These limitations highlight the need for robust contingency planning to optimize its potential in disaster scenarios.

## 6. Conclusion

**Figure 2:**
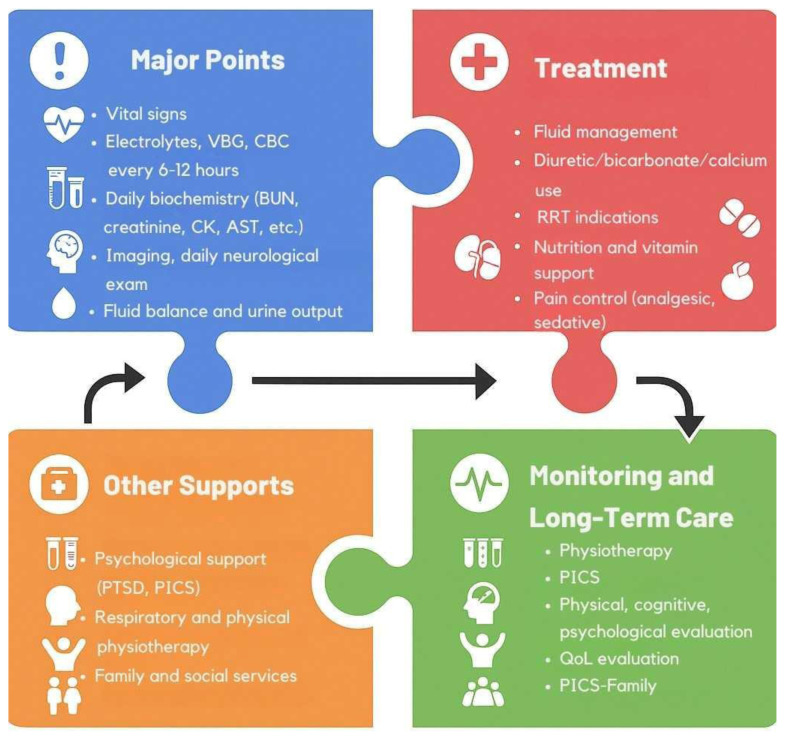
Schematic approach to the monitoring, management, and long-term follow-up of critically ill patients following earthquake disasters. VBG: venous blood gas, CBC: complete blood count, BUN: blood urea nitrogen, CK: creatinine kinase, AST: aspartate aminotransferase, RRT: renal replacement therapy, PTSD: posttraumatic stress disorder, PICS: postintensive care syndrome, QoL: quality of life This illustration was generated by ChatGPT 4.o and Canva.

The management of critically ill patients following earthquakes necessitates a multidisciplinary approach, encompassing swift fluid resuscitation, infection prevention, and evidence-based supportive care. Addressing both immediate and long-term needs is essential for improving survival rates and patient outcomes. Comprehensive monitoring and tailored interventions for complications such as CS, compartment syndrome, and coagulopathy can mitigate the risks of MOF and other sequelae. A stepwise schematic representation of these key elements—from early assessment and monitoring to long-term follow-up—is presented in Figure 2, aiming to support clinical decision-making and highlight the continuum of care required in such complex scenarios.

Future preparedness should focus on equipping healthcare professionals with disaster-specific training, strengthening healthcare infrastructure, and ensuring the availability of essential medical supplies. The integration of telemedicine and advanced technologies in disaster response may facilitate improved coordination and access to specialist care. Furthermore, ongoing research and the development of evidence-based guidelines are critical to refining management strategies for earthquake-related injuries. Multidisciplinary collaboration and systemic readiness are the cornerstones for optimizing care in disaster scenarios.

The February 6, 2023, earthquakes in southeastern Türkiye not only highlighted the devastating human toll of such disasters but also exposed critical vulnerabilities in emergency preparedness and critical care delivery. These events must catalyze systemic change, prompting governments, institutions, and the global medical community to prioritize resilience, foster international cooperation, and invest in scalable, adaptable response systems.

## Figures and Tables

**Table 1:** Prophylactic and preemptive antibiotic treatment scheme.

Situation	Treatment and duration
Injury or fasciotomy leading to tissue loss	Clindamycin 3 × 600 mg (5 days)
Soil contamination	Ciprofloxacin* 2 × 400 mg plus clindamycin 3 × 600 mg (5–7 days)
Corrected QT interval > 450 ms	Ceftazidime or cefepime plus clindamycin
Perforation in the abdomen	Ceftriaxone 1 × 2 g or ciprofloxacin* 2 × 400 mg IV plus metronidazole 3 × 500 mg IV
Penetrating injuries to the chest wall	Clindamycin 3 × 600 mg IV

IV: intravenous,

* Dose adjustment according to renal function if necessary.

Attention should be paid to QT prolongation in arrhythmic and especially elderly patients.

**Table 2:** Management principles of critically ill patients affected by the earthquake [66].

Crucial points	Recommendations
**Follow-up**	Electrolytes, venous blood gas (note lactate and BE), CBC every 6–12 h Calculation of SID and corrected anion gap Daily BUN, creatinine, albumin, uric acid, CK, myoglobin, LDH, AST, ALT, total bilirubin, DIC parameters CRP, PCT, prealbumin at admission Hourly volume balance Urine color tracking Extremity examination every 12 h for 6 P’s control Daily neurological examination Completed whole-body imaging
**Fluids**	Consider the extent of the damage, BSA, body and environmental temperature, age, CHF, and CKD history After extrication, 500–1000 cc/h (10–15 cc/kg) IV isotonic saline Taper fluid 50% after 2 h Evaluated 6 h later Anuria→500–1000 cc more than daily fluid loss 3–6 L/day if close monitoring is impossible Crystalloids→Avoid fluids containing potassium for the initial resuscitation Balanced crystalloids→Maintenance fluid preference according to potassium level (hypernatremia, hyperchloremic acidosis after isotonic saline) Burn or similar skin damage→Parkland formula of 4 mL × kg × burn percentage. Half in the first 8 h, the rest in 16 h Urine output target→1–3 cc/kg/h, up to 300 cc/h Hypervolemia→Remove excess fluid
**Diuretics**	Routine use is not recommended **Mannitol;** ○ With IV fluid therapy, no single-use ○ Contraindicated in oligoanuria ○ >20 cc/h urine output needed ○ 1–2 g/kg/day (maximum 120 g/day), 5 g/h→Added to hourly fluids ○ No >30–50 cc/h urine output→Discontinue ○ Attention→Electrolyte abnormalities; Na >150, osmolality >320 mOsm **Loop diuretics;** ○ Elderly? and hypervolemic patients ○ Deresuscitation needs→ARDS
**Bicarbonate**	To increase urine pH to >6.5 200–300 meq→First 24 h Prevent alkalosis→pH > 7.5, HCO3 > 30 mEq/dL Attention→Accumulation of calcium phosphate, hypernatremia, volume loading, hypocalcemia Ionized calcium follow-up
**Calcium**	Only in symptomatic hypocalcemia Empirically→Cardiac protection in severe hyperkalemia Its effect lasts 30–60 min Attention→Might lead to hypercalcemia during crush recovery
**RRT**	Oliguria, anuria Severe uremia (BUN >100 mg/dL, creatinine >8 mg/dL), hyperkalemia (>7 mmol/L), metabolic acidosis (pH < 7.1) Prophylactic/earlier IHD→Rapidly rising potassium CRRT→Hemodynamically unstable patients Hyperinflammatory state→Cytokine removal
**Infections**	Strict infection control measures Tetanus vaccination Antitetanus serum→Iron stinging or high soil contamination **Prophylactic or preemptive antibiotics** Fasciotomy Penetrating head, chest, and abdominal trauma Head fractures Open face fractures
**Analgesia/sedation**	Paracetamol, maximum dose is 4 g/day, attention→Hepatic failure Alternative→Tramadol, morphine (SC-IV), fentanyl (patch-IV) Attention→GIS hypomotility Good alternative→Ketamine (IM-IV) Regional block NSAIDs avoided
**Nutrition**	Potassium-poor CKD diet→30–35 kcal/kg/day Attention→Refeeding syndrome Thiamine, multivitamin and trace elements No protein restriction on RRT 1.5–2 g/kg/day protein→ IHD or CRRT
**Physiotherapy**	Early mobilization→According to orthopedics or other surgical departments Daily taken out of bed ROM exercises Respiratory physiotherapy Attention→Atelectasis due to pain
**Psychological/social supports**	Acute stress disorder→First month Risk of suicide→ Should not be left alone Posttraumatic stress disorder→After 1 month Prolonged grief Delirium risk Psychiatric and social service consultations
**After discharge**	Physiotherapy→Continue after discharge PICS→Physical, cognitive, and psychological evaluation Quality of life evaluation Burnout of families/caregivers→PICS-family Attention→PTSD

BE: base excess, CBC: complete blood count, SID: strong ion difference [Formula = (sodium + potassium) − (chloride + lactate)], BUN: blood urine nitrogen, CK: creatinine kinase, LDH: lactate dehydrogenase, AST: aspartate aminotransferase, ALT: alanine aminotransferase, DIC: Disseminated intravascular coagulopathy, CRP: C-reactive protein, PCT: procalcitonin, 6 P’s: pain, pressure, paresthesia, paresis-paralysis, paleness, pulselessness, BSA: body surface area, CHF: congestive heart failure, CKD: chronic kidney disease, IV: intravenous, Na: sodium, ARDS: acute respiratory distress syndrome, HCO3: bicarbonate, RRT: renal replacement therapy, IHD: intermittent hemodialysis, CRRT: continuous renal replacement therapy, SC: subcutaneous, GIS: gastrointestinal system, NSAID: nonsteroid antiinflammatory drug, PICS: postintensive care syndrome, PTSD: posttraumatic distress disorder
